# New anti-cancer chemicals Ertredin and its derivatives, regulate oxidative phosphorylation and glycolysis and suppress sphere formation in vitro and tumor growth in EGFRvIII-transformed cells

**DOI:** 10.1186/s12885-016-2521-9

**Published:** 2016-07-19

**Authors:** Sonoko Atsumi, Chisato Nosaka, Hayamitsu Adachi, Tomoyuki Kimura, Yoshihiko Kobayashi, Hisashi Takada, Takumi Watanabe, Shun-ichi Ohba, Hiroyuki Inoue, Manabu Kawada, Masakatsu Shibasaki, Masabumi Shibuya

**Affiliations:** Laboratory of Oncology, Institute of Microbial Chemistry, 3-14-23 Kamiosaki, Shinagawa-ku, 141-0021 Tokyo Japan; Numazu Branch, Institute of Microbial Chemistry, Miyamoto, Numazu-shi, Shizuoka Japan; Laboratory of Synthetic Organic Chemistry, Institute of Microbial Chemistry, Kamiosaki, Shinagawa-ku, Tokyo Japan; Institute of Physiology and Medicine, Jobu University, Takasaki-shi, Gunma Japan

**Keywords:** Ertredin, EGFRvIII, Sphere, 3D, Anchorage-independent, Oxidative phosphorylation, Glycolysis, Apoptosis

## Abstract

**Background:**

*EGFRvIII* is a mutant form of the epidermal growth factor receptor gene *(EGFR)* that lacks exons 2–7. The resulting protein does not bind to ligands and is constitutively activated. The expression of *EGFRvIII* is likely confined to various types of cancer, particularly glioblastomas. Although an anti-EGFRvIII vaccine is of great interest, low-molecular-weight substances are needed to obtain better therapeutic efficacy. Thus, the purpose of this study is to identify low molecular weight substances that can suppress EGFRvIII-dependent transformation.

**Methods:**

We constructed a new throughput screening system and searched for substances that decreased cell survival of NIH3T3/EGFRvIII spheres under 3-dimensional (3D)-culture conditions, but retained normal NIH3T3 cell growth under 2D-culture conditions. In vivo activity was examined using a mouse transplantation model, and derivatives were chemically synthesized. Functional characterization of the candidate molecules was investigated using an EGFR kinase assay, immunoprecipitation, western blotting, microarray analysis, quantitative polymerase chain reaction analysis, and measurement of lactate and ATP synthesis.

**Results:**

In the course of screening 30,000 substances, a reagent, “Ertredin” was found to inhibit anchorage-independent 3D growth of sphere-forming cells transfected with *EGFRvIII* cDNA. Ertredin also inhibited sphere formation in cells expressing wild-type *EGFR* in the presence of EGF. However, it did not affect anchorage-dependent 2D growth of parental NIH3T3 cells. The 3D-growth-inhibitory activity of some derivatives, including those with new structures, was similar to Ertredin. Furthermore, we demonstrated that Ertredin suppressed tumor growth in an allograft transplantation mouse model injected with *EGFRvIII*- or wild-type *EGFR*-expressing cells; a clear toxicity to host animals was not observed. Functional characterization of Ertredin in cells expressing *EGFRvIII* indicated that it stimulated EGFRvIII ubiquitination, suppressed both oxidative phosphorylation and glycolysis under 3D conditions, and promoted cell apoptosis.

**Conclusion:**

We developed a high throughput screening method based on anchorage-independent sphere formation induced by *EGFRvIII*-dependent transformation. In the course of screening, we identified Ertredin, which inhibited anchorage-independent 3D growth and tumor formation in nude mice. Functional analysis suggests that Ertredin suppresses both mitochondrial oxidative phosphorylation and cytosolic glycolysis in addition to promoting EGFRvIII degradation, and stimulates apoptosis in sphere-forming, EGFRvIII-overexpressing cells.

**Electronic supplementary material:**

The online version of this article (doi:10.1186/s12885-016-2521-9) contains supplementary material, which is available to authorized users.

## Background

Epidermal growth factor receptor (EGFR) expression and signaling are known to contribute to the development of multiple epithelial malignancies, including glioblastoma, squamous carcinomas of the skin, and breast cancer [1–6]. Thus, *EGFR *mutations have important implications for drug resistance and prognosis [7–9]. Among these mutations, an intragenic deletion of exons 2 to 7 of the wild-type *EGFR* (*EGFRwt*) gene results in an in-frame deletion of 267 amino acid residues from the extra-cellular domain of the 175-kDa EGFRwt. This deletion results in a truncated, about 140-kDa constitutively activated mutant EGFR, with an intracellular domain identical to EGFRwt [10–12]. This mutant *EGFR* gene was discovered by Shibuya et al. in 1988 [10, 13] and named ⊿*EGFR*, *de2-7 EGFR*, or more commonly, *EGFRvIII*. The *EGFRvIII* gene has been found in glioblastoma, lung, breast, ovarian, colorectal, head and neck squamous cell carcinoma (HNSCC), and prostate cancer. EGFRvIII signaling has been shown to correlate with a poor prognosis [14, 15]. There is extensive evidence indicating that EGFRvIII is a tumor-specific protein [15], and aberrant EGFRvIII signaling has been shown to be important in tumor progression. Because it is expressed only in tumor cells, it appears to be a rational and attractive target for cancer therapy [2, 15, 16]. Although the anti-EGFRvIII vaccine has received increased attention, it may not be effective for all EGFRvIII tumor-carrying patients. Therapies involving vaccines are difficult to apply in immune-suppressed cancer patients, and have potential risks such as the induction of autoimmune diseases. Thus, low-molecular-weight substances are required for efficient therapy.

Normal cells that grow in the adherent state undergo apoptosis shortly after losing their adhesion to the substratum, a phenomenon known as “anoikis” [13, 17–20]. However, cancer cells are still able to survive and grow in the absence of adhesion or anchorage to a substratum [12]. For example, glioblastoma cells overexpressing *EGFRwt* or *EGFRvIII* have been shown to be anchorage-independent. This “anchorage independence” is believed to be one of the most important oncogenic properties of cancer cells and cancer stem cells [19–22].

In the present study, we describe a high throughput method for the screening of EGFRvIII-cascade inhibitors. By screening 30,000 substances, we identified “Ertredin derivatives” that suppressed anchorage-independent growth in vitro and tumor growth in EGFRvIII-transformed cells.

## Methods

### Cell culture

NIH3T3 cell lines overexpressing human *EGFRvIII* (*de2-7EGFR*) (NIH3T3/EGFRvIII) and wild-type *EGFR* (NIH3T3/EGFRwt) were established using a previously reported method [10]. NIH3T3, NIH3T3/EGFRvIII, and NIH3T3/EGFRwt cells were maintained in Dulbecco’s Modified Eagle Medium (DMEM) supplemented with 5 % FBS. All cells were cultured with 50 U/mL penicillin/streptomycin at 37 °C in a humidified atmosphere of 5 % CO_2_ and 95 % air. Viable cell counts were assessed using the CellTiter 96 AQueous One Solution Cell Proliferation Assay or the CellTiter-Glo Luminescent Cell Viability Assay (Promega, Madison, USA).

### Materials

AG1478 was purchased from Wako (Osaka, Japan), gefitinib was obtained from AstraZeneca, erlotinib was from ChemieTek (Indianapolis, USA), and KT5720 and LY294002 were from Sigma-Aldrich. The chemical library included 30,000 low-molecular weight compounds supplied by the ChemBridge Screening Libraries (San Diego, CA, USA). Rabbit anti-human EGFR (D38B1), rabbit anti-phospho-EGFR Tyr1068 (D7A5), mouse anti-ubiquitin (P4D1), and HIF-1α monoclonal antibodies were purchased from Cell Signaling Technology (Danvers, USA). Mouse anti-β-actin (AC-15) monoclonal antibody was obtained fom Abcam (Cambridge, UK). Peroxidase-conjugated anti-rabbit and anti-mouse secondary antibodies were supplied by Jackson Immunoresearch (West Grove, PA, USA).

### Anchorage-independent 3D cell culture and screening

For the “3D cell culture,” 100 μL of a 2 × 10^5^ cells/mL solution was seeded on Corning Ultra-Low attachment surface (ULAS) plates (Corning, USA) and cultured for 3 days. In the course of screening for a 3D growth inhibitor, inhibition rate was calculated using the following equation 1:

$$ \mathrm{Inhibition}\ \mathrm{rate}\ \mathrm{of}\ \mathrm{N}\mathrm{I}\mathrm{H}3\mathrm{T}3/\mathrm{EGFRvIII}\ 3\mathrm{D}{\textstyle \hbox{-}}\mathrm{growth}\ \mathrm{b}\mathrm{y}\ \mathrm{a}\ \mathrm{chemical}=1 - \left(\mathrm{a}-\mathrm{b}\right)/\left(\mathrm{c}-\mathrm{b}\right) $$

where a = number of NIH3T3/EGFRvIII cells that survived upon treatment in 3D-culture conditions, b = number of NIH3T3 cells that survived in 3D-culture conditions, c = NIH3T3/EGFRvIII cells that survived with vehicle treatment in 3D-culture conditions.

In the course of screening for the “2D cell culture,” 100 μL of NIH3T3 (2–5 × 10^4^ cells/mL) were seeded per well of a 96-well tissue culture-treated plate. We calculated the inhibition rate of the NIH3T3 2D-culture growth with the following equation 2:

$$ \mathrm{Inhibition}\ \mathrm{rate}\ \mathrm{of}\ \mathrm{normal}\ \mathrm{N}\mathrm{I}\mathrm{H}3\mathrm{T}3\ 2\mathrm{D}{\textstyle \hbox{-}}\mathrm{growth}\ \mathrm{b}\mathrm{y}\ \mathrm{a}\ \mathrm{chemical}=1 - \left(\mathrm{d}/\mathrm{e}\right) $$

where d = number of NIH3T3 cells that survived with treatment in 2D-culture conditions, and e = number of NIH3T3 cells that survived with vehicle treatment in 2D-culture conditions.

In the 3D culture, viable cells were quantitated with the CellTiter-Glo luminescent cell viability assay (Promega). In the 2D culture, cell number was assessed by CellTiter 96 (Promega).

### Knockdown assays

Cells (2 × 10^5^ cells/mL, 10 mL/well) were seeded on ULAS 6-well plates for 3D growth, or were seeded on 12-well cell culture plates at 5 × 10^4^ cells/well for 2D growth. The cells were then transfected for 72 h (for 3D culture) or 24 h (for 2D culture) with 12 pmoles of small interfering RNA (siRNA) using Lipofectamine RNAiMAX transfection reagent (Life Technologies). After transfection, the cells were harvested and lysed for western blotting.

Cells (2 × 10^5^ cells/mL, 0.1 mL) were seeded on ULAS 96-well plates for 3D growth, or were seeded in 96-well cell culture plates at 2 × 10^3^ cells/well for 2D growth. The cells were then transfected for 72 h with 0.12 pmoles siRNA, and cell proliferation was quantitated. NM005228_stealth_2438 (RNA-GGAUCCCAGAAGGUGAGAAAGUUAA), NM005228_stealth_2858 (RNA-CAGAAGGAGGCAAAGUGCCUAUCAA), and M005228_stealth_2874 (RNA-GCCUAUCAAGUGGAUGGCAUUGGAA) were purchased from Life Technologies for EGFR-siRNA. Negative control siRNA (46-2002; Life Technologies) that did not target any mRNA sequence was used as a control.

### Western blotting

After washing with PBS, cells were treated with lysis buffer [50 mM HEPES (pH 7.2), 10 % glycerol, 150 mM NaCl, 1 % TritonX-100, 1 mM EGTA, 100 M PMSF, 100 units/mL of aprotinin, 10 mM Na_3_VO_4_, 100 mM NaF] [23]. Protein abundance in the cell lysate was assessed with the BCA Protein Assay Kit-Reducing Agent Compatible (Thermo Fisher Scientific, Waltham, U.S.A.). After addition of one-third the volume of the NuPAGE LDS Sample Buffer (4X) (Life Technologies) to the cell lysate, an appropriate amount of protein from the mixture was applied to 10 % SDS-PAGE. The proteins in the gel were transferred to a PVDF membrane using iBlot (Life Technologies). The protein was detected using a primary antibody and peroxidase-conjugated secondary antibody, and visualized with the ECL Prime Western Blotting Detection Reagent. The images were acquired using the ImageQuant LAS 4000 Mini (GE Healthcare Life Sciences) and processed by Adobe Photoshop.

### Ertredin derivatives

E1 (original Ertredin), E7, and E8 (a new substance), were synthesized as shown in Additional file 1. E2 was obtained from Enamine (Kiev, Ukraine), and E3, E5, and E15 were from Life Chemicals (Niagara-on-the-Lake, Canada). E4 and E14 were obtained from Pharmeks (Moscow, Russia), E6 was from UkrOrgSynthesis (Kiev, Ukraine), and E9-13 were from ChemBridge Screening Libraries.

### Mouse transplantation model

NIH3T3/EGFRvIII (1 × 10^5^) cells or NIH3T3/EGFRwt (1 × 10^6^) cells were suspended in 100 μL of 62.5 % v/v Matrigel (BD Biosciences) in DMEM and transplanted subcutaneously into female Balb/c nude mice at 7–8 weeks of age. Either Ertredin derivatives or gefitinib were dissolved in a vehicle consisting of saline, 10 % w/w DMSO, and 1 % w/w Tween80. After cell transplantation, 30 mg/kg Ertredin or I-Ertredin was injected intraperitoneally once daily from day 1–17. Gefitinib at 100 or 200 mg/kg was orally administrated to mice transplanted with these cells as a positive control.

Five to 6 mice were included in the vehicle-injected group, 4–5 in the Ertredin-or I-Ertredin-injected group, and 3–5 in the gefitinib-injected group. We measured the long and short diameters of the tumors every 2 or 3 days, and calculated tumor volume with the following equation: tumor volume = (long diameter) × (short diameter)^2^ / 2. On day 17 or 18, the tumors were extirpated, weighed, and photographed using a Nikon COOLPIX 990. The images were processed by Preview (MacOS). For TUNEL staining, parts of the tumors were fixed using 4 % paraformaldehyde phosphate buffer solution (Wako), followed by paraffin embedding.

### RT-PCR analysis

Total RNA templates for cDNA synthesis were isolated from 5 mg tumor tissue homogenate by using NucleoSpinRNAXS (Macherey-Nagel, Düren, Germany). cDNA synthesis was performed using the SuperScript Vilo cDNA Synthesis Kit (Thermo Fisher Scientific Inc.). For the differential RT-PCR products of EGFRvIII or EGFRwt, we used a forward primer sequence corresponding to an element in exon 1, and reverse primer sequence in exon 11 [24]. For common RT-PCR products, a forward primer sequence corresponding to an element in exon 13, and reverse primer sequence corresponding to exon 21 were used [25]. The primers were supplied by Sigma-Aldrich. AccuPower PCR PreMix (Bioneer, Daejeon, Korea) was used in PCR assays and the reaction proceeded under the following conditions: 94 °C for 2 min, 50 °C for 30 min, 30 cycles at 94 °C for 30 s, 60 °C for 60 s, and 72 °C for 30 s. After PCR by iCycler (Bio-Rad Laboratories, Hercules, U.S.A.), the reaction mixture was applied to 2 % agarose gel for electrophoresis with ethidium bromide. The bands were detected using an UV light trans-illuminator.

### Caspase activity

Cellular caspase activity was assessed by using the Caspase-Glo 3/7 Assay (Promega). The assay was performed according to the manufacturer’s instructions, and the detection of caspase-3/7 cleavage of the luminogenic substrate containing the DEVD sequence was developed using the ARVO SX1420 multi-label counter (Perkin Elmer).

### TUNEL (TdT-mediated dUTP nick-end labeling) staining

TUNEL staining of spheres was carried out essentially by method described previously [26, 27]. Briefly, cells (3 × 10^5^ cells/mL) were seeded on 100-mm ultra-low attachment dishes; after 3 days, the spheres were harvested, washed with PBS, and fixed with 4 % paraformaldehyde phosphate buffer solution followed by paraffin embedding. Thin sections from the paraffin block were assayed using the TUNEL method with an apoptosis in situ detection kit (Wako).

### In vitro EGFR kinase assay

Direct inhibition of EGFR kinase by Ertredin and AG1478 was investigated with the Kinase Enzyme System (Promega). In this assay, we used the C-terminal kinase recombinant protein of human EGFR expressed in SF9 cells for the enzyme, and poly-(Glu4, Tyr1) for the substrate, as described in the manufacturer’s protocol.

### Ubiquitination assay

Cells (3 × 10^5^ cells/mL) were seeded on 10 cm ULAS (Ultra-Low attachment surface) dishes and cultured overnight. Sphere formation was observed, and MG132, a proteasome inhibitor, was added to the medium. After 2 h of cultivation, the indicated chemical agents were added. After cultivation for 17 h, the spheres were harvested and washed with PBS, followed by suspension in lysis buffer. Cells in the suspension were disrupted via repeated passages through a 23-gauge syringe. Cellular debris was pelleted by centrifugation at 10,000 × g for 10 min at 4 °C. The protein in the supernatant was quantitated, and a primary antibody was added to 1 mg/mL of cellular protein suspension at a 1/100 dilution. After 1 h of incubation at 0 °C, 20 μL of Protein A/G PLIUS-Agarose (Santa Cruz Biotechnology, Inc. Dallas, USA) was added to each mixture and the mixture was incubated at 4 °C overnight with mixing. The agarose beads were collected by centrifugation at 1000 × *g* for 30 s at 4 °C and washed 3 times with 1 mL lysis buffer. After the final wash, the beads were resuspended in sample buffer and the suspension was applied to 10 % SDS-PAGE for western immunoblot analysis after boiling for 5 min.

### ATP synthesis in mitochondria

Cells under 2D-cell culture conditions (2000) were seeded in 384-well plates with the galactose-supplemented culture medium (10 mM galactose, 0 mM glucose, 0 % serum) and cultured for 1 h. After the cells attached to the bottom of each well, the test substances were added. Cells under 3D-cell culture conditions (20,000) were seeded on in 96-well Clear Black Round Bottom ULAS plates (Corning) and cultured for 4 days. The medium was then changed to galactose-supplemented culture medium after washes; the cells were then cultured for 1 h, followed by the addition of test substances for 2 h. ATP was measured using the Mitochondrial ToxGloAssay (Promega).

### Microarray analysis

The total RNA was isolated from cells by NucleoSpinRNA (Macherey-Nagel, Düren, Germany), according to the manufacturer’s instructions. RNA samples were quantified using an ND-1000 spectrophotometer (NanoDrop Technologies, Wilmington, U.S.A.) and the quality was confirmed with an Experion System (Bio-Rad Laboratories). The cRNA was amplified, labeled, and hybridized to a 60 K Agilent (Santa Clara, U.S.A.) 60-mer oligomicroarray, according to the manufacturer’s instructions. All hybridized microarray slides were scanned by an Agilent scanner. Relative hybridization intensities and background hybridization values were calculated using Agilent Feature Extraction Software (9.5.1.1). The raw signal intensities of two samples were log2-transformed and normalized by quantile algorithm with ‘preprocessCore’ library package [28] on Bioconductor software [29]. To identify up- or down-regulated genes, we calculated Z-scores [30] and ratios (non-log scaled fold-change) from the normalized signal intensities of each probe for comparison between the control and experimental sample. We then established criteria for the regulated genes: up-regulated genes had a Z-score ≥ 2.0 and ratio ≥ 1.5-fold, down-regulated genes had a Z-score ≤ −2.0 and ratio ≤ 0.66.

### Quantitative PCR

Total RNA templates for cDNA synthesis were isolated from approximately 2–3 × 10^6^ cells by using the NucleoSpinRNAII (Macherey-Nagel). cDNA synthesis was performed using the SuperScript Vilo cDNA Synthesis Kit. Quantitative PCR was performed using the SYBR Premix Ex Taq™ II (TAKARA, Kusatsu, Japan) and Thermal Cycler Dice Real Time System (TAKARA). The standard protocol (95 °C for 30 s, for one cycle; 95 °C for 5 s and 60 °C for 30 s, for 40 cycles; 95 °C for 15 s, 60 °C for 30 s, 95 °C for 15 s, for one cycle) was used for all reactions. Relative quantification was carried out with glucuronidase beta (Gusb) as an endogenous control. The primer sets were as follows: GLUT1 (NM_011400.3, forward: TGTGGGCATGTGCTTCCAGTA, reverse: GCCTTTGGTCTCAGGGACTTTG), HK1 (NM_001146100.1, forward: AGAGGCCTAGACCACCTGAATGTAA, reverse: ACTGTTTGGTGCATGATTCTGGAG), HK2 (NM_013820.3, forward: GGCCAACTTCATGGACAAGCTAC, reverse: CCACGCCACTGGACTTGAAC), PFK1 (NM_008826.4, forward: CAGTCCGGTCACAGAACTCAAG, reverse: GCATCAGCCGCAGATTCA), PKM (NM_001253883.1, forward: ATGCCTGGGCTGAGGATGTC, reverse: ACTACACGCATGGTGTTGGTGAA), LDHA (NM_001136069.2, forward: GAACTGGGCACTGACGCAGA, reverse: CCAATGGCCCAGGATGTGTA), PDK1 (NM_172665.5,forward: GGAAGTCCATCTCATCGAAAGCA, reverse: AAAGCCGCCTAGCGTTCTCA), MCT1 (NM_009196.4, forward: GGCTTGGTGACCATTGTGGA, reverse: TGATGCCCATGCCAATGAA), GUSB (NM_010368.1, forward: CTGTGACCGATACGGGATTGTG, reverse: ACCTCTAGGTGGTGCCGAAGTG).

### Lactate Assay

Cells (2 × 10^6^) were seeded in ULAS 6 well plates, and test substances were added after 1 day of culture. After 1 day of treatment, the cells were harvested, and lactate was quantitated by L-Lactate Assay Kit (Abcam).

## Results and discussion

### Sphere formation and growth of cells in 3D-culture induced by *EGFRvIII* expression

**Fig. 1:**
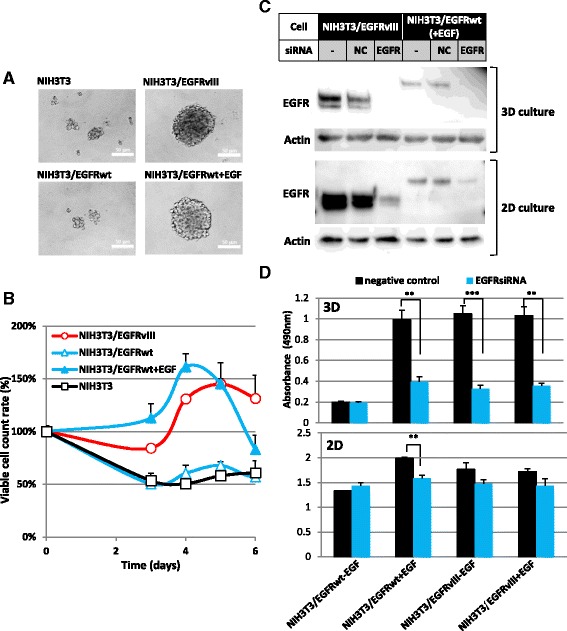
Overexpression of *EGFRvIII* or *EGFRwt* induced anchorage-independent growth of NIH3T3 cells. **a** Anchorage-independent (3D) growth of NIH3T3/EGFRvIII or NIH3T3/EGFRwt cells. Cells (2 × 10^5^ cells/mL) were seeded and observed by phase-contrast microscopy after cultivation for 3 days. Images were acquired using an Olympus DP71 microscope camera and processed by Adobe Photoshop. **b** Growth curves of NIH3T3 cells overexpressing *EGFRvIII* or *EGFRwt* in 3D cell cultures. Cells (1 × 10^5^ cells/mL, 100 μl) were seeded on ULAS 96-well plates on day 0. Viable cells were counted at the indicated times by CellTiter-Glo Luminescent Cell Viability Assay. **c** *EGFR*-siRNA inhibited EGFRvIII and EGFRwt protein expression in NIH3T3 cells overexpressing each protein. Cells were lysed and 6 μg protein was applied to 10 % SDS-PAGE gel. Cells were cultured on ULAS plate for 3 days (3D) or on a normal cell-attachment plate for 1 day (2D) after *EGFR*-siRNA transfection. **d** *EGFR*-siRNA inhibited NIH3T3/EGFRvIII and NIH3T3/EGFRwt anchorage-independent 3D growth. Viable cell counts were measured by CellTiter 96 AQueous One Solution Cell Proliferation Assay 3 days after transfection with *EGFR*-siRNA (NM005228_stealth_2438). Cells were cultured with (50 ng/mL) or without EGF on ULAS plates (upper, 3D) or on normal tissue culture plate (lower, 2D)

We used EGFRvIII cDNA-transfected NIH3T3 cell as an in vitro model. Using this model, we could compare the inhibitory potency of substances against EGFRvIII-driven-3D-sphere formation and anchorage-dependent growth of parental normal cells. We seeded NIH3T3/EGFRvIII cells on ULAS plates to investigate anchorage-independent 3D growth; the cells formed spheroidal clumps more than 100 μm in diameter in the wells after 3 days of culture (Fig. 1a, upper right), whereas the control NIH3T3 cells did not (Fig. 1a, upper left). NIH3T3/EGFRwt cells also formed spheres when they were cultured with EGF (Fig. 1a, lower right), but not without EGF (Fig. 1a, lower left). The number of viable NIH3T3/EGFRvIII cells forming spheres in the ULAS plates began to increase 3 or 4 days after seeding (Fig. 1b). The number of NIH3T3/EGFRwt cells cultured with EGF also increased, and then decreased more quickly (Fig. 1b). The conditions of cells in the inner core regions of the sphere are reported to include hypoxia and low nutrition [31]. In addition, the internalized EGF-EGFRwt complex is degraded inside the cells, and the medium becomes deprived of EGF under 3D-sphere-cultural conditions. These conditions may decrease the number of viable NIH3T3/EGFRwt cells at 5 or 6 days after seeding, as shown in Fig. 1b. The number of NIH3T3/EGFRwt cells seeded in ULAS plates in the medium without EGF decreased in a manner similar to control NIH3T3 cells (Fig. 1b). In contrast, EGFRvIII kinase is constitutively active without the ligand, and thus the survival signal appears to be transduced in a relatively stable manner [32], and the decrease in cell counts may not occur as rapidly. However, these differences between NIH3T3/EGFRwt cells and NIH3T3/EGFRvIII cells were not pivotal in our screening assay because the number of surviving cells was measured on day 3. Next, we investigated whether the expression of *EGFRvIII* or *EGFRwt* is essential for anchorage-independent 3D sphere formation. An *EGFR*-siRNA targeting both *EGFRvIII* and *EGFRwt* strongly suppressed these proteins (Fig. 1c) and significantly decreased 3D sphere formation in both EGFRvIII and EGFRwt cell cultures with EGF (Fig. 1d, upper). However, a similar effect was not observed in 2D-culture (Fig. 1d, lower). The analysis using other *EGFR*-siRNAs (NM005228_stealth_2858 or NM005228_stealth_2874) gave essentially the same results. These results strongly suggest that *EGFRvIII* and *EGFRwt* expression is essential for sphere formation and anchorage-independent 3D growth of NIH3T3/EGFRvIII and NIH3T3/EGFRwt cells, respectively.

### Throughput screening for inhibitors suppressing anchorage-independent growth induced by EGFRvIII

**Fig. 2:**
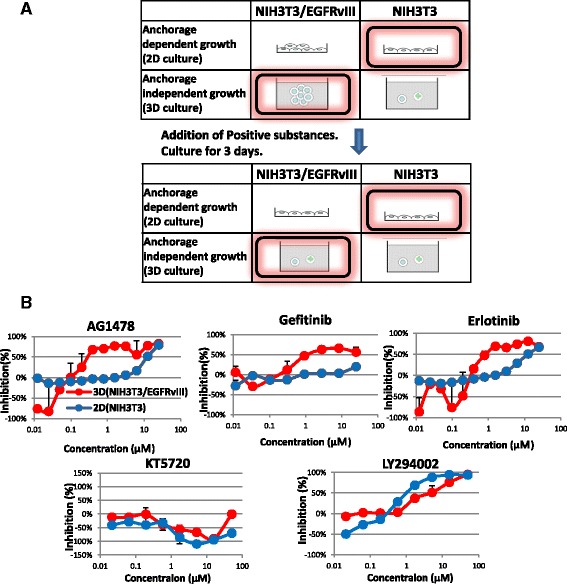
High throughput screening system developed to identify inhibitors of anchorage-independent growth induced by EGFRvIII. **a** Outline of the screening system. **b** Potency of protein kinase inhibitors in the screening systems. NIH3T3/EGFRvIII cells were seeded on ULAS 96-well plates for 3D-culture (red line). NIH3T3 cells were seeded on normal tissue culture plates for 2D-culture (blue line). Each inhibitor was added to the medium on day 0. Three days after inoculation, viable cells were counted and the extent of inhibition was calculated

To isolate new inhibitors of the EGFRvIII cascade, we developed a screening system to identify substances that suppress the cell survival of NIH3T3/EGFRvIII spheres under 3D-culture conditions, but retained normal NIH3T3 growth under 2D-culture conditions (Fig. 2a). To examine the validity of this system, we used AG1478, gefitinib, and erlotinib (EGFR kinase inhibitors), KT5720 (PKA inhibitor), and LY294002 (PI3 kinase inhibitor). As shown in Fig. 2b, AG1478, gefitinib and erlotinib efficiently suppressed cell survival of NIH3T3/EGFRvIII spheres in 3D-culture at 1 μM, but not NIH3T3 cell survival in 2D-culture up to 10 μM in our system. These EGFR kinase inhibitors were assigned as “positive hits” in our assay system. KT5720 had no effect on cell numbers, whereas LY 294002 suppressed cells in both 3D and 2D-culture conditions. LY294002 did not specifically inhibit the anchorage-independent growth of EGFRvIII, as it also suppressed NIH3T3 cells in 2D culture. Therefore, both the PKA inhibitor and the PI3 kinase inhibitor were excluded in our system, and this assay system is well suited for our purposes.

### Ertredin derivatives inhibited NIH3T3/EGFRvIII cells in 3D culture

**Fig. 3:**
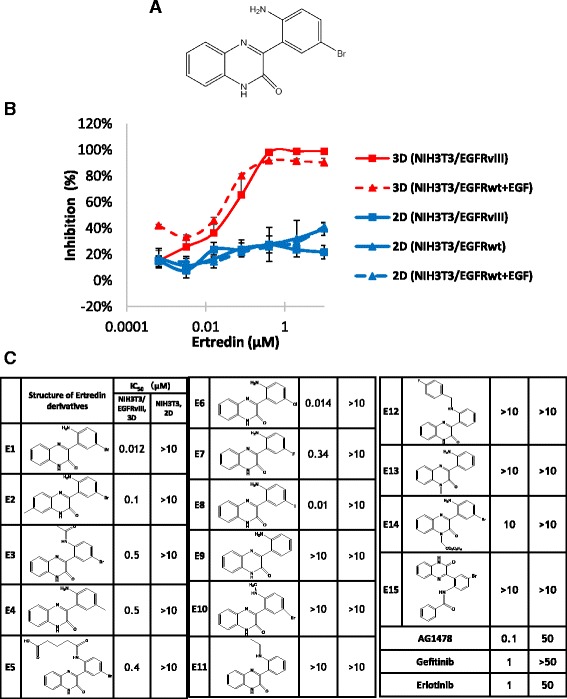
Ertredin derivatives inhibited anchorage-independent growth of NIH3T3/EGFRvIII cells. **a** Structure of Ertredin. **b** Inhibitory activity of Ertredin against 3D growth of NIH3T3/EGFRvIII (red solid line) or NIH3T3/EGFRwt (red dashed line), and 2D growth of NIH3T3. Cells were seeded and cultured on ULAS plates (3D) or normal tissue culture plates (2D). Extent of inhibition was detected on day 3. EGF (100 ng/mL) was added to NIH/3 T3/EGFRwt 3D culture. **c** Structure-activity relationships of Ertredin derivatives

To isolate new inhibitors of EGFRvIII, we carried out a screening assay using a chemical library containing 30,000 low-molecular weight compounds (Fig. 2). Through this screening, we identified only one positive hit for 3-(2-amino-5-bromophenyl)-2 (1H)-quinoxalinone (Fig. 3a). We named this substance Ertredin, derived from “**E**GF **r**eceptor **t**h**re**e **d**imensional **in**hibitor.” Ertredin significantly inhibited 3D sphere growth of NIH3T3/EGFRvIII cells and NIH3T3/EGFRwt cells in the presence of EGF, but it did not inhibit 2D growth of NIH3T3, NIH3T3/EGFRvIII, or NIH3T3/EGFRwt cells (Fig. 3b).

Ertredin (E1 in Fig.3c), its fluoro-analog (E7), and a novel iodo-derivative (E8) named I-Ertredin, were synthesized and used for biological study. All the synthetic compounds demonstrated 3D sphere-inhibitory activity similar to the original Ertredin. With regard to 2D growth, none of the Ertredin derivatives produced cytotoxicity in NIH3T3 cells (Fig. 3c). When the Br located in the phenyl ring was substituted by Cl (E6 in Fig. 3c), or I (E8), 3D inhibitory potency was retained, whereas fluorination (E7) or methylation (E4) decreased the activity, and hydrogenation (E9) removed inhibitory activity completely.

The inhibitory activities of E2, E3, E5, and E10-12 on NIH3T3/EGFRvIII cell growth in 3D-culture was remarkable; these compounds differ from Ertredin (E1) in the amino groups of their phenyl ring. When the secondary amine was changed to a tertiary amine (E13, E14), the inhibitory activity in 3D-culture was no longer observed.

The original compound Ertredin was previously reported to inhibit mDia-1-mediated actin assembly by targeting mDia1 FH2 at IC50 = 2.5 μM in vitro [33]. However, there are no published data showing the effect of the compound on mDia-1 protein on the cell level; the concentrations required for in vitro inhibition of mDia1 activity were very high, in the order of 2 μM. Moreover, replacing 5-Br with Cl or F in this compound was reported to remove the suppression activity on mDia-1 [33] while retaining the inhibitory activity of these compounds on anchorage-independent growth (Fig. 3c). Therefore, the activity of this compound (Ertredin) on mDia1 suppression observed in vitro may be unrelated to its inhibitory activity on anchorage-independent growth for the cells shown here.

### Tumor suppression by Ertredin in vivo

**Fig. 4:**
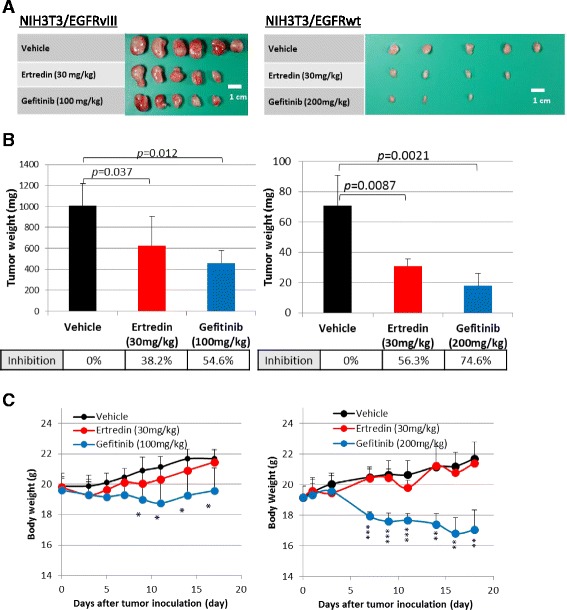
Ertredin suppressed NIH3T3/EGFRvIII (left) and NIH3T3/EGFRwt (right) tumorigenicity in nude mice. **a** Macroscopic tumor appearance at day 17 (NIH3T3/EGFRvIII) and day 18 (NIH3T3/EGFRwt). **b** Weights of tumors at day 17 (NIH3T3/EGFRvIII) and day 18 (NIH3T3/EGFRwt). **c** Time-dependent changes in body weights of mice transplanted with NIH3T3/EGFRvIII (left) and NIH3T3/EGFRwt. *P* values in two-tailed paired student *t*-test are shown: *p < 0.05; **p < 0.01; ***p <0.001

As shown in Fig. 4a (left, upper line), NIH3T3/EGFRvIII cells are tumorigenic in athymic nude mice, while the parental NIH3T3 cells are not (data not shown). NIH3T3/EGFRwt cells were also tumorigenic in athymic nude mice, but tumor size was smaller than that of NIH3T3/EGFRvIII cells (Fig. 4a, right, upper line), most likely due to a limited EGF level in vivo [34].

Ertredin treatment by intraperitoneal injection at 30 mg/kg body weight/day efficiently suppressed tumorigenicity equal to that of a positive control, gefitinib, which was administered orally at 100 mg/kg/day or 200 mg/kg/day (Fig. 4b). After injection with Ertredin, the mice carrying tumors did not lose body weight, suggesting no clear toxicity to host animals (Fig. 4c). On the other hand, the tumor-bearing mice treated with gefitinib did lose body weight (Fig. 4c). As shown in Additional file 2, I-Ertredin also suppressed the tumorigenic activity of NIH3T3/EGFRvIII cells without remarkable toxicity. In these tumors transplanted with NIH3T3/EGFRvIII or NIH3T3/EGFRwt cells, we confirmed the expression of *EGFRvIII* or *EGFRwt* mRNAs using RT-PCR (Additional file 3).

### Ertredin induced apoptosis but did not inhibit EGFR kinase

**Fig. 5:**
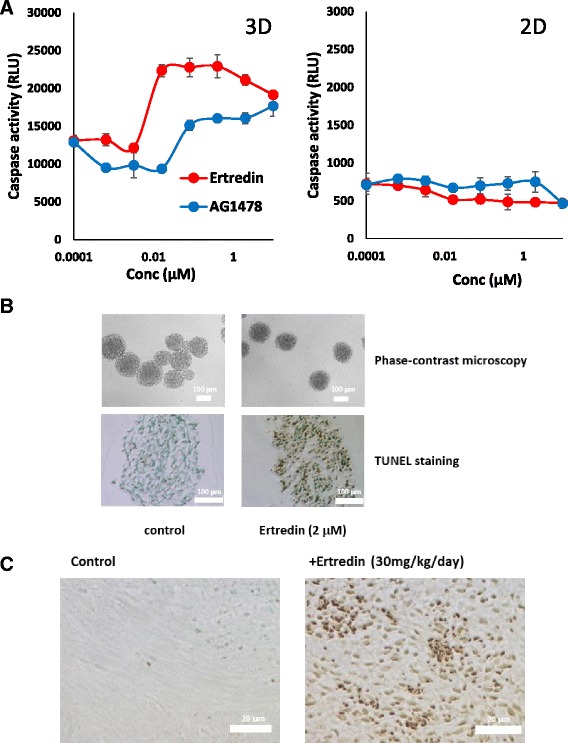
Ertredin induced apoptosis in vitro 3D-sphere and in vivo tumors of NIH3T3/EGFRvIII cells. **a** Cells with the addition of Ertredin (red line) or AG1478 (blue line) were seeded on ULAS plates (left) or tissue culture treated plates (right), followed by cultivation for 72 h. Caspase 3/7 activities were measured as described in Materials and Methods. **b** (Upper) Spheres were observed by phase contrast microscopy 72 h after seeding of cells on ULAS plates. (Lower) Apoptotic cells (brown) in paraffin sections were determined by TUNEL assay. Cells were cultured in medium with Ertredin (2 μM) (right) or vehicle (left). Apoptotic cells were stained brown. DNA counterstained with Methyl Green. **c** TUNEL staining for the detection of apoptotic cells in tumors isolated from mice treated with Ertredin (right) or control mice without Ertredin (left)

To examine the process of cell death as mediated by Ertredin, we quantitated the activity of the apoptosis effectors, caspase 3/7, within the cell. In NIH3T3/EGFRvIII cells, caspase 3/7 activities were increased when Ertredin was added at more than 0.01 μM under 3D culture conditions (Fig. 5a, left), while no effect on caspase activity was seen in 2D culture with Ertredin (Fig. 5a, right). The up-regulation of caspase activity in 3D culture alone was also observed after the addition of AG1478. We further examined apoptosis induction by TUNEL staining both spheres and tumors. TUNEL-positive cells were observed in about 70 % of the spheres formed in the medium with Ertredin, whereas they were observed in less than 5 % of the spheres without Ertredin (Fig. 5b, lower). TUNEL-positive cells were often observed in tumors isolated from mice treated with Ertredin (Fig. 5c, right), while they were rarely observed in those without Ertredin (Fig. 5c, left).

**Fig. 6:**
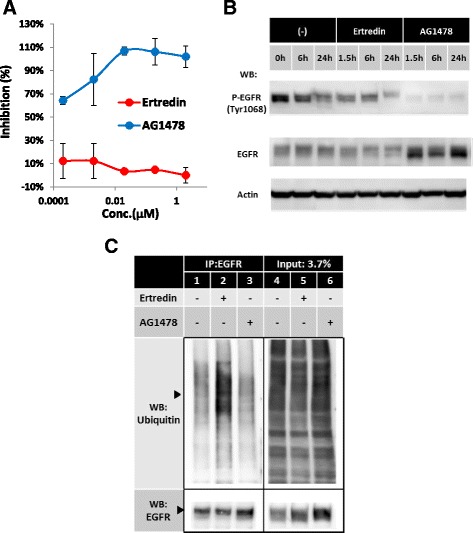
Ertredin did not directly inhibit EGFR kinase but stimulated ubiquitination of EGFR in NIH3T3/EGFRvIII cells. **a** Direct effect of Ertredin (red line) or AG1478 (blue line) on the tyrosine kinase of human EGFR. **b** Effect of Ertredin on autophosphorylation of EGFRvIII in NIH3T3/EGFRvIII cells. Cells (3 × 10^5^ /mL, 5 mL) were seeded on ULAS 6-well plates and cultured for 22 h. Ertredin or AG1478 was then added to a final concentration of 1 μM. Cells were harvested at the indicated time after the addition of chemicals. Each cell lysate (16 μg of protein) was applied on 10 % SDS-PAGE followed by immunoblot analysis. **c** NIH3T3/EGFRvIII spheres in ULAS dishes were treated with Ertredin (final concentration, 2 μM), AG1478 (final concentration, 2 μM), or vehicle for 17 h in the presence of MG132 (50 μM). Lysates were prepared from the cells and subjected to immunoprecipitation with anti-EGFR antibody followed by western blot with respective antibodies as shown (lane 1–3). Input lanes indicate 3.7 % of lysate used in immunoprecipitation (lane 4–6). The triangles indicate the estimated position of EGFRvIII

Next, we investigated whether Ertredin directly inhibited the kinase activity of EGFRvIII, similar to AG1478. The EGFRvIII protein possesses an in-frame deletion of 267 amino acid residues from the extra-cellular domain, but retains the kinase domain of wild-type EGFR. Therefore, we examined the inhibitory activity of Ertredin against recombinant wild-type hEGFR kinase domain in vitro. As shown in Fig. 6a, Ertredin did not directly inhibit EGFR kinase, but the activity was strongly suppressed by AG1478 as expected. With NIH3T3/EGFRvIII cells, 50 % of EGFRvIII autophosphorylation was abrogated by the addition of Ertredin, and this coincided with a decrease in protein abundance (Fig. 6b). In contrast, AG1478 completely inhibited EGFRvIII autophosphorylation, and the protein abundance was not decreased.

It is known that the EGFRvIII protein is ubiquitinated by Cbl and degraded in proteasome-dependent manner, similar to activated EGFR [35, 36]. Ertredin was found to up-regulate ubiquitination of EGFRvIII via anti-hEGFR immunoprecipitation (Fig. 6c, lane 2), compared to control cells or cells treated with AG1478 (lane 3). The amount of EGFRvIII after Ertredin treatment decreased to one-half of the control, however, ubiquitination of total cellular proteins was unaltered with or without Ertredin (lane 4–5). These results suggest that digestion of EGFRvIII in proteasomes is activated in cells treated with Ertredin, but in a limited manner. Ertredin suppressed 3D cell growth and induced a remarkable level of apoptosis at concentrations less than 0.1 μM; however, only 50 % inhibition of the EGFRvIII protein level was observed even at 1 μM. Thus, targets for Ertredin, other than the ubiquitination and degradation pathway, may exist within EGFRvIII-cells in 3D-culture.

### Ertredin suppresses mitochondrial oxidative phosphorylation and cytosolic glycolysis

We carried out further screening in other sources, including 30,000 microorganism metabolites. Through this screening, we found that Piericidin and other Streptomyces metabolites suppressed the anchorage-independent growth of EGFRvIII-overexpressing cells (data not shown). Piericidin is known to be an NADH dehydrogenase inhibitor. Some of other hits also showed potent mitochondrial inhibition. Respiratory chain inhibitors were previously shown to inhibit a 3D culture of T47D breast cancer cells [37]. Therefore, we examined the effect of Ertredin on oxidative phosphorylation. We also investigated the suppressive potency of Rotenone and Antimycin against NIH3T3/EGFRvIII 3D growth, which are well known to be respiratory chain complex I and complex III inhibitors, respectively.

**Fig. 7:**
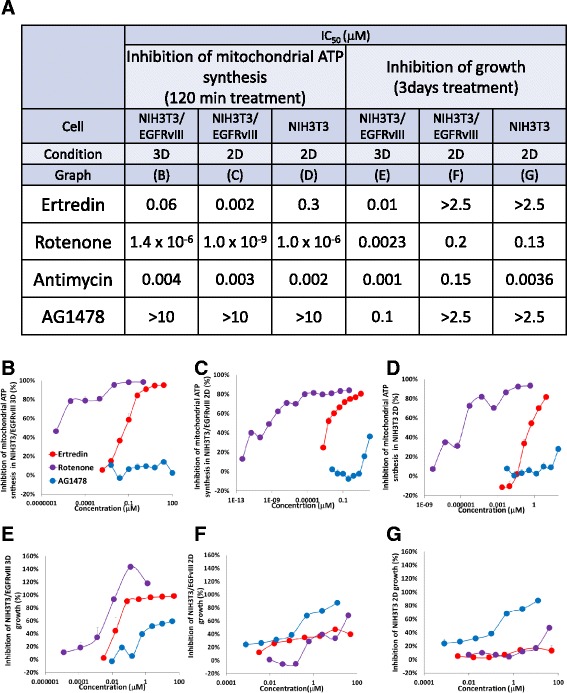
Ertredin inhibited mitochondrial ATP synthesis in NIH3T3/EGFRvIII cells under 3D conditions. **a** Summary of the assay results. Inhibition of mitochondrial ATP synthesis by 120 min treatment with Ertredin (red line), Rotenone (purple line), AG1478 (blue line) in **b** NIH3T3/EGFRvIII cells under 3D-culture conditions, **c** NIH3T3/EGFRvIII cells under 2D-culture conditions, and **d** NIH3T3 cells under 2D-culture conditions. The cells were cultured in absence of glucose (in galactose-containing medium) and then ATP synthesis was measured. Inhibition of cell growth with the addition of the substances for 3 days in **e** NIH3T3/EGFRvIII cells under 3D-culture conditions, **f** NIH3T3/EGFRvIII cells under 2D-culture conditions, and **g** NIH3T3 cells under 2D-culture conditions

We observed the inhibitory activity of Ertredin against oxidative phosphorylation in NIH3T3/EGFRvIII cells by measuring short-time ATP synthesis in the absence of glucose (galactose-containing medium) (Fig. 7a). This inhibitory effect was more effective on 2D-cultured NIH3T3/EGFRvIII cells than 3D-cultured cells (30 times at the IC_50_ value). Similar to Ertredin, Rotenone also had a greater effect on the 2D-cultured NIH3T3/EGFRvIII than 3D- cultured cells (1400 times at the IC_50_ value). The mechanism underlying the difference in sensitivity to Ertredin between 3D- and 2D- cultured NIH3T3/EGFRvIII cells is not well understood at this moment, but one hypothesis is that 3D-sphere conditions signal the Warburg effect machinery, and decrease its sensitivity to an exogenous mitochondrial inhibitors.

By comparing Fig. 7b–d with e–g, we can see a similarity between the suppression of sphere growth and the decrease in mitochondrial ATP synthesis by Ertredin in NIH3T3/EGFRvIII under 3D conditions. On the other hand, the growth under 2D conditions was not affected by suppression of the mitochondrial ATP synthesis. These results suggest that the inhibition of mitochondrial ATP synthesis is important for the inhibition of growth in NIH3T3/EGFRvIII under 3D conditions.

**Fig. 8:**
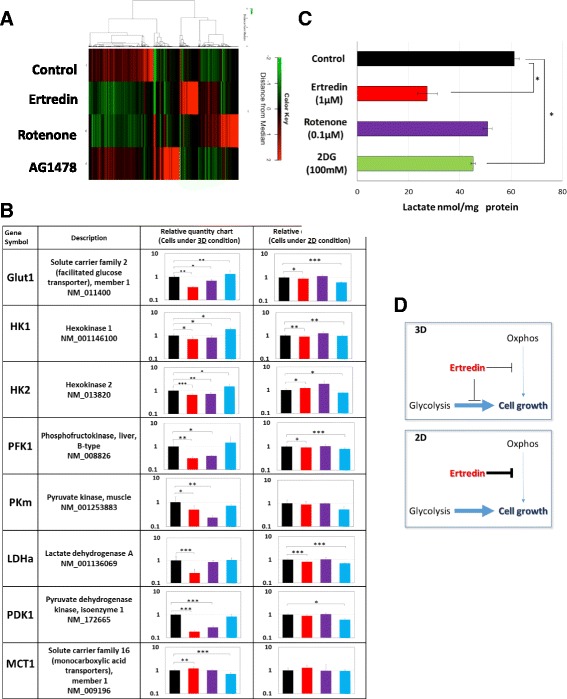
Ertredin suppresses the expression of glycolytic pathway enzymes in NIH/3 T3 EGFRvIII cells under 3D conditions. NIH3T3/EGFRvIII cells (2 × 10^6^ cells/well) were seeded on a ULAS 6-well plate for 3D culture and 10 cm tissue culture-treated plate for 2D cell culture. Ertredin (2 μM), Rotenone (1.25 μM) AG1478 (2 μM) or DMSO (0.1 %) was added to the medium and after 24 h of culture, the cells were harvested and total RNA was isolated. **a** Microarray heat map. **b** Relative quantitative charts obtained by qPCR analysis for each glycolysis pathway gene in cells treated with Ertredin (red bar), Rotenone (purple bar), AG1478 (blue bar), and DMSO (black bar) under 3D and 2D conditions. **c** Lactate in the cells cultured with the indicated substances for 24 h under 3D conditions. 2-deoxyglucose (2DG), a glucose analogue, is known to inhibit hexokinase, the first step of glycolysis. **d** An explanatory diagram of cell-growth inhibition under 3D conditions by Ertredin

To examine this functional similarity, we performed microarray analyses of the gene expression profiles of 3D spheres treated with Ertredin, Rotenone, and the tyrosine kinase inhibitor, AG1478 (GEO ID; GSE76959). Interestingly, the heat map indicated partial analogy between Ertredin and Rotenone, but not between Ertredin and AG1478 (Fig. 8a). Indeed, many glycolytic-pathway enzymes were significantly decreased in the cells treated with Ertredin and Rotenone, but not AG1478. Using qPCR, we more closely examined the modifications in the gene expression of glycolytic pathway enzymes. As shown in Fig. 8b, under 3D conditions, Ertredin and Rotenone both suppressed Glut1, PFK1, PKm, and PDK1. The LDHa was significantly suppressed by Ertredin but not by Rotenone. As shown in Fig. 8c, lactate was remarkably decreased in Ertredin-treated spheres, which is consistent with the results shown in Fig. 8b. A hexokinase inhibitor, 2DG, also decreased lactate, but its efficacy was weaker than Ertredin. It has been previously indicated that the accumulation of lactate in tumor cells is a pivotal and early event in the development of malignancies [38]. Inhibition of tumorigenicity by Ertredin may be related to the suppression of lactate levels in NIH3T3/EGFRvIII cells.

We also examined the gene expression of glycolytic pathway enzymes under 2D conditions. Surprisingly, neither Ertredin nor Rotenone suppressed the expression of glycolysis-related enzymes at detectable levels (Fig. 8b). Thus, these results indicate that the suppression of the gene expression in glycolysis enzymes with Ertredin occurs specifically under 3D conditions.

Glycolytic enzymes are known to be positively regulated by hypoxia-inducible factor (HIF)-1α; thus, we investigated HIF-1α levels in cells treated with Ertredin under 3D and 2D conditions (Additional file 4). Western blotting indicated that HIF-1α was not decreased in cells treated with Ertredin under 3D conditions. In contrast, Ertredin and Rotenone slightly decreased HIF-1α under 2D conditions. These results suggest that Ertredin suppresses the expression of glycolytic pathway genes independently from HIF-1α under 3D conditions.

We demonstrated that Ertredin suppressed both mitochondrial ATP synthesis and cytosolic glycolysis under 3D conditions. Ertredin may efficiently inhibit total energy metabolism. Both Ertredin and Rotenone suppress mitochondrial ATP synthesis and the gene expression of many glycolysis enzymes in 3D-cultured cells. These results suggest that at least one of the targets of Ertredin within the cell is very similar to that of Rotenone. Ertredin inhibits mitochondrial ATP synthesis under both 2D and 3D conditions but it suppresses cell survival and growth under 3D-culture conditions compared to 2D conditions. A possible explanation is firstly that Ertredin suppresses glycolytic pathway preferentially under 3D conditions (Fig. 8d), and secondly that 3D-sphere growth is more sensitive to mitochondrial inhibition as shown previously (33).

Ertredin was found to be safe and capable of suppressing tumors in mice. The mechanical analysis suggests that Ertredin is multifunctional. Inhibition of glycolysis by Ertredin may suppress the Warburg effect, a common metabolic alteration of most tumor cells. And almost total inhibition of energy metabolism by Ertredin may initiate apoptosis in cancer cells without affecting normal cells. Because Ertredin has these unique characteristics, it could potentially lead to the development of new cancer therapeutic agents. We are currently studying the effect of Ertredin and its derivatives on human tumors.

## Conclusion

We introduced a high throughput method in the identification of inhibitors of anchorage-independent growth of NIH3T3/EGFRvIII cells. Over the screening of 30,000 substances, we identified Ertredin that inhibited the growth of allograft tumors in vivo. Ertredin promoted apoptosis in 3D-spheres as well as tumors of mice transplanted with NIH3T3/EGFRvIII cells. Functional characterization revealed that Ertredin suppressed not only mitochondrial oxidative phosphorylation but also cytosolic glycolysis under 3D-culture conditions. In addition, an increase in EGFRvIII ubiquitination and degradation were observed in NIH3T3/EGFRvIII cells treated with Ertredin. Our evidence suggests that the multi-effects of Ertredin induce apoptosis in NIH3T3/EGFRvIII cells under 3D-culture conditions and tumors in vivo.

## Abbreviations

2D, 2-dimensional; 2DG, 2-Deoxy-D-glucose; 3D, 3-dimensional; DMSO, Dimethyl sulfoxide; EGFR, epidermal growth factor receptor; EGFRvIII, EGFR variant III; EGFRwt, wild-type EGFR; FBS, Fetal bovine serum; HIF, Hypoxia-inducible factors; PBS, phosphate buffered saline; PI3 kinase, Phosphoinositide 3-kinase; PKA, protein kinase A; RT-PCR, reverse transcriptase-polymerase chain reaction; SDS-PAGE, sodium dodecyl sulfate-polyacrylamide gel electrophoresis; siRNA, small interfering RNA; TUNEL, TdT-mediated dUTP nick-end labeling; ULAS, Ultra-Low attachment surface.

## Additional files

Additional file 1: Organic synthesis methods. (DOCX 46 kb) Additional file 2: I-Ertredin suppressed NIH3T3/EGFRvIII tumorigenicity. NIH3T3/EGFRvIII cells were subcutaneously transplanted into nude mice. The administration schedule was as described in Fig. 4 legend. (A) Weights of tumors. (B) Time-dependent changes in body weight of mice. (PDF 297 kb) Additional file 3: Tumors isolated from mice transplanted with NIH3T3/EGFRvIII or NIH3T3/EGFRwt cells were overexpressing human EGFRvIII and EGFRwt, respectively. RNA was purified from tissue homogenates and RT-PCR was performed using primer sets (A): E1 forward and E11 reverse, (B): E13 forward and E21 reverse. Each primer was shown in (C): Primers. (PDF 250 kb) Additional file 4: Effect of Ertredin on HIF-1α protein levels under 3D or 2D conditions. Cells were cultured with the indicated substances for 24 h under 3D or 2D conditions. Sixteen micrograms of protein in each cell lysate were applied on SDS-PAGE and the HIF-1α protein level was estimated by western blotting. Dimethyloxaloylglycine (DMOG) is an inhibitor of prolyl hydroxylase (PHD) bringing that transports HIF-1α proteoasome systems. (PDF 235 kb)
