# Supplementary material for: New anti-cancer chemicals Ertredin and its derivatives, regulate oxidative phosphorylation and glycolysis and suppress sphere formation in vitro and tumor growth in EGFRvIII-transformed cells
Source: BMC Cancer. 2016 Jul 19;16:496. doi: 10.1186/s12885-016-2521-9 (PMC4949881; doi:10.1186/s12885-016-2521-9)
Supplement: Additional file 1: — Organic synthesis methods. (DOCX 46 kb) [file 12885_2016_2521_MOESM1_ESM.docx]

**Additional file 1**

**Organic synthesis methods**

General

The reactions were performed in an oven-dried test tube or round bottom flask with a Teflon-coated magnetic stirring bar unless otherwise noted. All work-up and purification procedures were carried out with reagent-grade solvents under ambient atmosphere. Infrared (IR) spectra were recorded on a JASCO FT/IR 4100 Fourier transform infrared spectrophotometer. NMR was recorded on JEOL ECA-600 spectrometers. Chemical shifts for proton are reported in parts per million downfield from tetramethylsilane and are referenced to residual protium in the NMR solvent (DMSO-*d*_6_: δ 2.49 ppm). For ^13^C NMR, chemical shifts were reported in the scale relative to NMR solvent (DMSO-*d*_6_: δ 39.7 ppm) as an internal reference. NMR data are reported as follows: chemical shifts, multiplicity (s: singlet, d: doublet, t: triplet, br: broad signal), coupling constant (Hz), and integration. High-resolution mass spectra (ESI-Orbitrap) were measured on ThermoFisher Scientific LTQ Orbitrap XL. Materials were purchased from commercial suppliers and were used without purification.

Synthesis of Ertredin derivatives


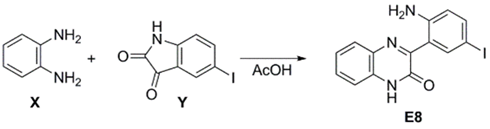
E1 and E7 were synthesized according to the reported procedure.^1^ All the spectral data were identical to the reported ones.

Synthesis of iodo-derivative of ertredin, E8.

3-(2-Amino-5-iodophenyl)-2(1*H*)-quinoxaline (E8)

5-Iodoisatin (81.9 mg, 0.30 mmol) and 1,2-phenylenediamine (32.4 mg, 0.30 mmol) were dissolved in 1.5 mL of AcOH, and the resulting solution was stirred at room temperature for 2 days. The mixture was concentrated in vacuo, and purified with silica gel column chromatography (n-hexane/AcOEt = 1/1) to give E8 (18.4 mg, 50.7 μmol) in 17% yield as an orange solid: mp 268–269 °C ; IR (KBr) ν 1660, 1562, 1482, 1211, 815, 755, 745 cm^-1^; HRMS (ESI) Anal. calcd. for C_14_H_11_IN_3_O m/z 363.9947 (M+H)^+^, found 363.9944; ^1^H NMR（600 MHz, DMSO-d_6_）：δ 6.64 (2H, brs), 6.65, (1H, d, *J* = 4.0 Hz), 7.28 (1H, dt, *J* = 4.0 Hz, 4.0 Hz, 0.5 Hz), 7.30 (1H, dd, *J* = 4. Hz 0, 0.5 Hz), 7.38 (1H, dd, *J* = 4.0 Hz, 1.0 Hz), 7.49 (1H, dt, *J* = 4.0 Hz, 4.0 Hz, 0.5 Hz), 7.79 (1H, dd, *J* = 4.0 Hz, 0.5 Hz), 8.41 (1H, d, *J* = 1.5 Hz), 12.50 (1H, brs, NH); ^13^C NMR（150.9 MHz, DMSO-d_6_）：δ 74.75, 115.41, 118.68, 120.04, 123.03, 128.09, 129.79, 131.30, 138.37, 138.99, 148.4, 154.16, 155.06.

1. Dowlatabadi R, Khalaj A, Rhaimian S, Montazeri M, Amini M, Shahverdi A, Mahjub E. Impact of substituents on the isatin ring on the reaction between isatins with ortho-phenylenediamine. Synth Comm 2011; 41: 1650-1658.
