# Supplementary material for: New anti-cancer chemicals Ertredin and its derivatives, regulate oxidative phosphorylation and glycolysis and suppress sphere formation in vitro and tumor growth in EGFRvIII-transformed cells
Source: BMC Cancer. 2016 Jul 19;16:496. doi: 10.1186/s12885-016-2521-9 (PMC4949881; doi:10.1186/s12885-016-2521-9)
Supplement: Additional file 2: — I-Ertredin suppressed NIH3T3/EGFRvIII tumorigenicity. NIH3T3/EGFRvIII cells were subcutaneously transplanted into nude mice. The administration schedule was as described in Fig. 4 legend. (A) Weights of tumors. (B) Time-dependent changes in body weight of mice. (PDF 297 kb) [file 12885_2016_2521_MOESM2_ESM.pdf]

Additional File 2

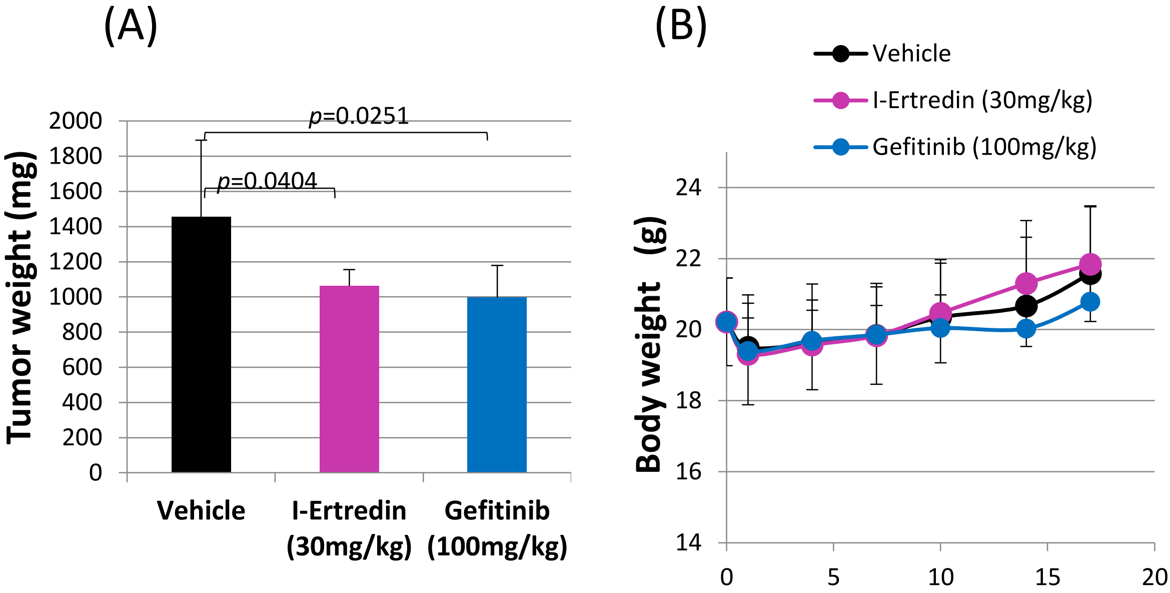

Inhibition	0%	26.9%	31.5%

File name:Additional File 2

File format:.PDF

Title of data:
I-Ertredin suppressed NIH3T3/EGFRvIII tumorigenicity.

Description of data:
NIH3T3/EGFRvIII cells were subcutaneously transplanted into nude mice. The administration schedule was as described in Fig. 4 legend. (A) Weights of tumors. (B) Time-dependent changes in body weight of mice.
