# Supplementary material for: New anti-cancer chemicals Ertredin and its derivatives, regulate oxidative phosphorylation and glycolysis and suppress sphere formation in vitro and tumor growth in EGFRvIII-transformed cells
Source: BMC Cancer. 2016 Jul 19;16:496. doi: 10.1186/s12885-016-2521-9 (PMC4949881; doi:10.1186/s12885-016-2521-9)
Supplement: Additional file 3: — Tumors isolated from mice transplanted with NIH3T3/EGFRvIII or NIH3T3/EGFRwt cells were overexpressing human EGFRvIII and EGFRwt, respectively. RNA was purified from tissue homogenates and RT-PCR was performed using primer sets (A): E1 forward and E11 reverse, (B): E13 forward and E21 reverse. Each primer was shown in (C): Primers. (PDF 250 kb) [file 12885_2016_2521_MOESM3_ESM.pdf]

Additional File 3

(A)

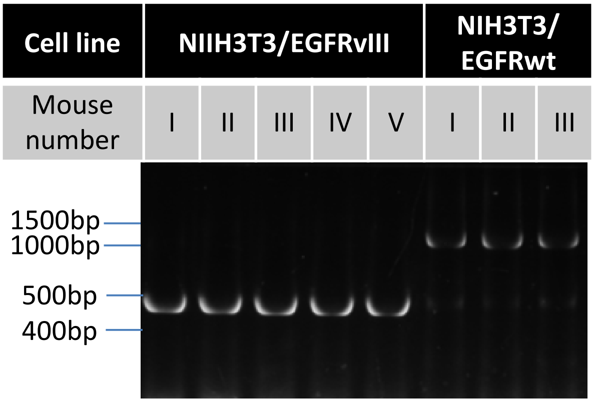

(B)

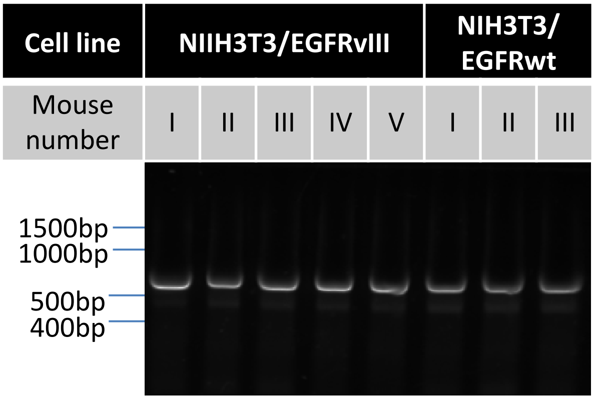

(C)

Primer set	Forward primer	Reverse primer	Estimated product length (bp)	
			NIH3T3/ EGFRwt	NIH3T3/ EGFRvIII
**E1-E11**	TCTTCGGCTAGCAACGATGCGACCCTC	GCCGCGTATGATTTCTAGATTCTCAAAGGC	1299	448
**E13-E21**	TGCGTCTCTTGCCGGAAT	TTGATAGCGACGGGAATTTTAAC	667	667

File name :Additional File 3

File format:.PDF

Title of data:
Tumors isolated from mice transplanted with NIH3T3/EGFRvIII or NIH3T3/EGFRwt cells were overexpressing human EGFRvIII and EGFRwt, respectively.

Description of data:
RNA was purified from tissue homogenates and RT-PCR was performed using primer sets (A): E1 forward and E11 reverse,(B): E13 forward and E21 reverse. Each primer was shown in (C): Primers.
