# Supplementary material for: New anti-cancer chemicals Ertredin and its derivatives, regulate oxidative phosphorylation and glycolysis and suppress sphere formation in vitro and tumor growth in EGFRvIII-transformed cells
Source: BMC Cancer. 2016 Jul 19;16:496. doi: 10.1186/s12885-016-2521-9 (PMC4949881; doi:10.1186/s12885-016-2521-9)
Supplement: Additional file 4: — Effect of Ertredin on HIF-1α protein levels under 3D or 2D conditions. Cells were cultured with the indicated substances for 24 h under 3D or 2D conditions. Sixteen micrograms of protein in each cell lysate were applied on SDS-PAGE and the HIF-1α protein level was estimated by western blotting. Dimethyloxaloylglycine (DMOG) is an inhibitor of prolyl hydroxylase (PHD) bringing that transports HIF-1α proteoasome systems. (PDF 235 kb) [file 12885_2016_2521_MOESM4_ESM.pdf]

Additional File 4

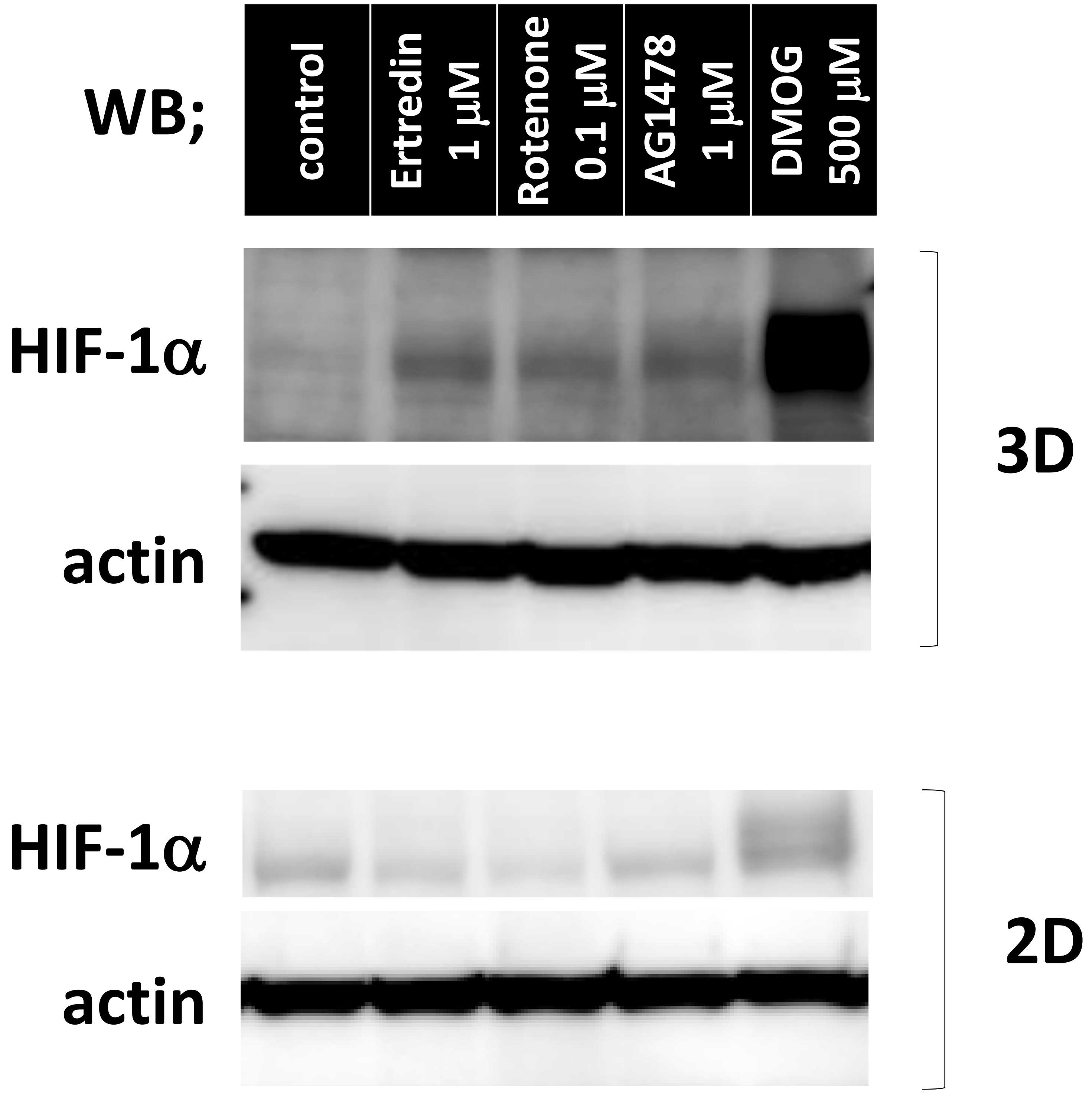

File name :Additional File 4

File format:.PDF

Title of data:
Effect of Ertredin on HIF-1α protein levels under 3D or 2D conditions.

Description of data:
Cells were cultured with the indicated substances for 24 h under 3D or 2D condition. Sixteen micrograms of protein in each cell lysate were applied on SDS-PAGE and the HIF-1α protein level was estimated by western blotting. Dimethyloxaloylglycine (DMOG) is an inhibitor of prolyl hydroxylase (PHD) bringing that transports HIF-1α proteoasome systems.
